# The implications of adults understanding events conveyed in American Sign Language as a function of language status

**DOI:** 10.3389/fpsyg.2026.1904654

**Published:** 2026-10-13

**Authors:** Joseph P. Magliano, Christopher A. Kurby, Jessica Scott, Peter K. Crume, Justin T. Malone, Monique D. Johnson, Tonya Gibson, Tatyania Watts

**Affiliations:** 1 Department of Learning Sciences, Georgia State University, Atlanta, GA, United States; 2 Department of Psychology, Grand Valley State University, Grand Rapids, MI, United States

**Keywords:** American Sign Language (ASL), ASL learners, event cognition, narrative comrpehension, native signers

## Abstract

**Introduction:**

Signed languages convey events via multiple articulators (e.g., hands, face, shoulders, and mouth), which are used to convey situational information about the characters, their goals, emotions, interactions with characters and objects, and how they occur in a spatial–temporal framework. The present study was conducted to assess the sensitivity to changes in situational dimensions (e.g., characters, goals, emotions, space–time) and low-level visual change when deaf native users of American Sign Language (ASL) experienced stories conveyed in ASL.

**Methods:**

Native signers viewed videos of stories that were conveyed in ASL and engaged in an event segmentation task. A situational change score measured the number of changes in situational dimensions, and a visual change score was computed to measure the amount of changes in the movement of the signer.

**Results:**

The likelihood of making event segmentation judgments was positively predicted by situational change scores, but not visual change scores; nor was there an interaction between situational change and visual change scores. In contrast, a prior published study conducted the same experiment with learners of ASL and found that the relationship between situational change scores and segmentation judgments was moderated by perceptual change such that sensitivity to situational change decreased as visual change increased. A cross-experiment analysis revealed that segmentation judgments from native signers were more strongly predicted by situational change scores than learners of ASL, whereas the judgments from ASL learners were more strongly predicted by visual change scores than native signers.

**Conclusion:**

These results suggest that experience with a signed language may influence how viewers attend to and integrate situational information during event comprehension and provide implications for theories of event cognition and ASL learning.

## 1 Introduction

Learning signed languages, such as American Sign Language (ASL), as a second language has significant challenges for those whose first language is a spoken language (Emmorey et al., 2009; Hilger et al., 2015), and this is particularly the case when learning how to process events conveyed in signed languages (Crume et al., 2025). Understanding these challenges is important because ASL has become a popular second language in American universities and colleges (Robinson and Henner, 2018), with some students taking these courses with the goal to become professional ASL interpreters. While both signed and spoken languages share features (e.g., they have an embedded structure involving phonology, the lexicon, syntax, semantics, and discourse), the way events are conveyed in signed and spoken languages is very different. In spoken languages, most of the semantic content that enables listeners to access event knowledge is conveyed orally through the speech organs (Perniss and Özyürek, 2015), albeit gestures and other paralinguistic cues can contain event information (Goldin-Meadow et al., 2008). However, in signed languages there are multiple articulators that convey information in a three-dimensional space (Brentari, 2010; Emmorey et al., 2009; Supalla, 1986). An important aspect of the use of multiple articulators is the application of non-manual articulators (i.e., not involving signs produced by the hands) during signing. This includes embodied actions such as gesture, eye gaze, facial expression, head position and movement (Brentari et al., 2018; Goldin-Meadow and Brentari, 2017). Important event information is conveyed in the hand movements that comprise signs (Krebs et al., 2021), and in particular for verbs (Krebs et al., 2026). However, non-manual articulates also convey event information that supplements the sign(s) being used by the signer. For instance, a positive statement can be negated through a head shake, and a statement can become a question with the raising or lowering of eyebrows. Moreover, the hands, face, body position (e.g., positioning of the shoulders) all contain important event information regarding who is involved, where they are located in space, what they are doing (who is speaking and acting, which is called role shift), information about deixis and perspective, etc. (Liddell, 2003).

These differences can create challenges for those learning signed languages as a second language. First, learners of signed languages must acquire the lexical and morphological knowledge associated with the signs that comprise a signed language (Emmorey, 1995), which may not be dissimilar to acquiring knowledge of the orally produced sounds that make up the phonology and lexicon of a language (Petitto et al., 2016). Second, non-manual markers are an essential means of understanding sign communication, and can be challenging for learners of a signed language to recognize (Herrmann and Steinbach, 2013). Third, a challenge arises if learners assume that the majority of event information is conveyed through the hand articulators (Emmorey et al., 2009). If one attends primarily to the signs produced by the hands, then important event information will be missed from the non-manual articulators. A fourth challenge comes from the fact that important grammatical information is marked temporally in different places for languages in different modalities (Brentari, 2010). For example, in many spoken languages, such as English, verb modification indicates a change in tense, whereas in signed language, such as ASL, tense is usually marked at the beginning of the sentence, as ASL generally follows the word order of (time, topic, comment) (Brentari, 2010). Finally, the syntactic structure of sentences is conveyed in the three-dimensional space in signed languages that runs from top-to-bottom from the head to the waist, left-to-right from an extension of one arm to the other, and back-to-front from just behind the signer to arm’s length in front of the signer (referred to as signing space). This space is used to convey the structure of events that are described by marking subjects, objects, and actions in relationship to one another (Brentari, 2010; Uyechi, 1994). Grammatical-morphological information is important for the activation of knowledge in event schemas when events are described in language in spoken languages (e.g., Ferretti et al., 2007), and there is every reason to believe that the same is the case for signed languages.

### 1.1 Differences in attentional selection for ASL learners and native users

These challenges culminate in a problem of visual attentional selection (i.e., knowing where and when to look) for second language learners of signed languages (Crume et al., 2025). If second language learners attend to the hands, rather than other articulators, then they will not only miss important event information for specific events that are being conveyed, but it could hamper the timeframe of developing proficiency in a signed language relative to acquiring a new spoken language.

Emmorey et al. (2009) explored whether there were differences in attentional selection when processing short narratives or spatial description for Deaf native signers (i.e., Deaf individuals who were born into a signing family) and novice learners of ASL. They hypothesized that the learners would fixate on the hands of the signer more than native users, but that hypothesis was not substantiated. Both learners and native users primarily fixated on the face of the signer. However, learners fixated more on the signer’s mouth than native users. Emmorey et al. (2009) speculated that this happened because the learners were attending to mouth cues for the English words associated with the signs more than native users. Additionally, they found that learners shifted away from the signer’s face more than native signers. Emmorey et al. (2009) reasoned that learners had a smaller perceptual span than native users. In the context of reading, the perceptual span refers to the amount of text that is informationally useful in a single fixation. They extrapolated this context to refer to the amount of the three-dimensional space in which the signing occurred that was informationally useful. Learners had to fixate on other parts of the three-dimensional space to extract important event information. While they did not find evidence that learners fixate more on the hands, this study does demonstrate differences in attentional selection between learners and native signers. Additionally, the materials that they used were relatively short (less than 2 min in length) and are used in ASL instruction for second language learners and may not reflect the complexity of the events described in natural language exchanges.

Crume et al. (2025) conducted a study that also suggests that there may be challenges of attentional selection for second language learners of signed languages. The present study is an important follow-up study to Crume et al. (2025) and as such, we describe it in detail. They conducted a study involving students enrolled in an ASL interpreter program in a four-year university. This study was motivated by the event indexing model that assumes that understanding narratives events involves monitoring continuity along *situational dimensions*, such as characters, goals, emotions, causality, spaces, and time (Zwaan and Radvansky, 1998). The event indexing model assumes that when people perceive changes in these events, this then leads to an understanding that the events in the story have changed. Perceived changes in situational dimensions lead the situation model in episodic memory (i.e., the memory representation for the narrative) to be updated to reflect those changes. One approach that has been used to test this assumption is an event segmentation task (e.g., Magliano et al. (2012); Zacks et al., 2009) which involves having participants make *event segmentation judgments* whenever they perceived there to be changes in events that make up a larger event (e.g., story) (Newtson, 1973). For example, Magliano et al. (2012) had participants engage in an event segmentation task while viewing the same stories as either conveyed as wordless picture stories or in text. They did a *situational dimensions continuity analysis* to determine if each story unit (pictures or sentences) depicted changes in characters (i.e., introduced a new characters), characters’ goals (the beginning or completion of a goal), character emotions, causality (i.e., depicted an event that could not be explained by prior story events), and the spatial and time. They found that the likelihood of making an event segmentation judgment (i.e., perceiving a change in the events) was positively similarly positively predicted by changes in the situational dimensions similarly across text and picture versions of stories.

Crume et al. (2025) was inspired by Magliano et al. (2012). Adult learners of ASL watched videos of folktales being conveyed in ASL or English (orally). The ASL versions were created by a deaf native signer who translated the written English versions with the instruction to do so as closely as possible. To illustrate this aspect of the materials, Table 1 contains the first four sentences of *A Farmer’s Bride* and the corresponding ASL gloss for those sentences (i.e., a gloss is a propositional representation of content conveyed in ASL discourse). As can be seen the ASL gloss reflects the same story content as the English. However, there were differences: information in two English sentences was combined into one ASL sentence: The ASL story explicitly added the event of the farmer walking through his fields, which was implicit in the English version. Crume et al. (2025) performed a situational dimension continuity analysis of the folktales conveyed in ASL and English.^1^ Table 2 shows the situations that were identified in the continuity analyses, which involved space, time, characters, objects, character goals, character internal states, causal anomalies, character-character interactions, and character-object interactions. While watching the videos on a computer, participants engaged in event segmentation tasks in which they pressed the spacebar on a keyboard whenever they felt that the events in the story had changed. The times in the videos where they made their judgments were recorded. One second time bins for the videos were created. In those time bins, Crume et al. (2025) determined (1) if participants made event segmentation judgments (2) the number of situational dimensions that changed based on the situational dimension continuity analysis. They found that likelihood of making event segmentation judgments was higher when there were changes in the situational dimensions when processing the English folktales. Surprisingly, none of the situational dimensions were significant predictors of segmentation judgments in ASL.

**Table 1:** Sample ASL gloss translation of “*A Farmer’s Bride*.”

English	ASL GLOSS
Once there was a farmer who was very wealthy.	LONG-AGO RICH FARMER WEALTHY
He had everything he wanted except a wife.	HE HAD LONG-LIST THINGS, UNDERSTAND, NO WIFE, WANT WIFE WANT
	DV (Walking on his farm) FARM LARGE-PLOT PEOPLE WORKING
One day the farmer saw a young maid.	LOOK-AROUND SPOT SAW YOUNG WOMAN WORKING HIS FARM
She was working in a hayfield.	

The ASL gloss is a propositional representation of the event information present in the ASL translation of the English sentence. DV refers to depicted verb.

**Table 2:** Situational dimensions that were identified in the situational dimension continuity analysis.

Dimension	Description
Space	Introduction of a new narrative space or characters move to a different narrative space than prior events.
Time	Introduction of a new time frame or there is a break in narrative time from events in prior sentences.
Character	Introduction of new characters or reintroductions of old characters that were previously involved in the story but not yet in the current episode.
Object	Introduction of a new object.
Goal	The beginning of a new character goal or the ending of a previously established goal.
Internal state	Change in perceptual, cognitive, affective, or knowledge in character.
Causal anomalies	Story event occurs that cannot be explained by prior story context.
Character-Character interaction	Characters start interacting or there is change in an established character-character interaction.
Character-object interaction	Characters start to physically interact with objects.

Crume et al. (2025) conducted an exploratory analysis that was inspired by Emmorey et al.’s (2009) unsubstantiated hypotheses and the anecdotal experience of two of the authors (Crume and Malone) who are instructors in the interpreter training program regarding learners overly attending to the hands of signers. They postulated a *post hoc* hypothesis that if participants were attending closely to the hands, they would be more likely to make the judgments when the hands stopped moving. They conducted a visual analysis of the ASL videos to create a visual change score for each time bin, which was operationalized as the average amount of pixel by pixel change in the 1 s time bins. They reasoned that visual change and hand movement would be strongly positively correlated because most of the moment-to-moment visual change in the videos would be based on hand moments (i.e., they were the part of the signer’s body that was moving the most). They also created a situational change score, which was a composite of the number of situational dimensions changing in each time bin (which ranged from 0 to 6). They assessed if the visual change and situational changes scores were predictive of event segmentation judgments. Consistent with their hypothesis, the likelihood of making an event segmentation judgment increased as a function of the amount of visual change. Importantly, they found that there was an interaction between the situational change and the visual change scores: the situational change score was a significant positive predictor of event segmentation change scores, but that positive relationship declined as visual change scores increased. They interpreted this interaction as suggesting that the participants were attending to the motion of the signer and buffering the event segmentation judgments until they were less active.

### 1.2 Overview of the present study

Crume et al. (2025) noted that a replication of the study with native signers would bolster their interpretation of the data, and specifically if native signers’ segmentation judgments were not significantly predicted by visual change scores. The purpose of the present study is to provide that replication. This study makes use of data from a larger study involving native signers that used the same materials and methodology as Crume et al. (2025). The present study specifically replicates and extends the exploratory analysis regarding relative contributions of the situation change and visual change scores on the perception of event boundaries. We additionally conducted planned cross-experiment analysis to assess the relative sensitivity to changes in situational and visual change in learners and native signers when making segmentation judgments. This analysis yielded further insights into challenges for native users of spoken languages in acquiring signed languages.

The present study addressed the following research questions:

*Research Question (RQ) 1*: Are situational changes and visual change scores positively associated with event segmentation judgments for native signers?
*Research Question (RQ) 2*: Do visual change scores moderate the relationship between situational change scores and event segmentation judgments for native signers?
*Research Question (RQ) 3*: Are the segmentation relationships with situational change and visual change scores moderated by whether one is a native signer or learner of ASL?

It was hypothesized that native signers are sensitive to the situational information conveyed in multiple articulators. On the other hand, it was hypothesized that ASL learners focus on situational information conveyed by hand movements (Crume et al., 2025). These hypotheses led to distinct predictions regarding the relationships between situational change scores, visual change scores, and the likelihood of making event segmentation judgments for each Research Question. With respect to RQ1, situational change scores were predicted to be a significant, positive predictor of the likelihood of making an event segmentation judgment. With respect to RQ2, it was predicted that visual changes scores would not be a significant predictor of the likelihood of making event segmentation judgments. With respect to RQ3, it was predicted that language status would moderate the relationships between situational and visual change scores and the likelihood of making event segmentation judgments. Specifically, the slope for the relationship between situational change scores and the likelihood of perceiving an event boundary should be more steeply positive for native ASL users than ASL learners. Conversely, the slope for visual change scores should be more steeply positive for ASL learners than native ASL users.

## 2 Methods

As noted above, we replicated Crume et al. (2025) with deaf native ASL users. However, the present study uses only data collected from the ASL story condition. For the sake of clarity, the methods section below provides a full account of the methods of the larger study.

### 2.1 Participants

Twenty native signers participated in the study and were compensated $30 for their participation. Native signers are defined (1) Deaf or Hearing individuals who were born into a signing family and as such, the signed language was their first language or (2) are deaf individuals who were exposed to signed language prior to 6 years old and a signed language is their primary language. Though in most populations, acquisition of a language at age 5 or 6 would be considered late, most Deaf individuals are born to hearing parents without any knowledge of a signed language (Mitchell and Karchmer, 2005), and these children are most likely to begin acquiring ASL at school entry, around age 6. Fifteen of the participants were recruited from staff who worked at a Deaf school in Atlanta Georgia. The five remaining participants were recruited via flyers posted to Deaf community listservs and snowball sampling from the Atlanta metropolitan area. Twelve of the participants were deaf and 8 were hearing individuals born to Deaf parents. The average age of participants was 38. Thirteen of the participants identified as white, 6 identified as black, and 1 identified as Asian. Eleven participants identified as female, 8 identified as male, and 1 identified as non-binary. The present study also made use of the sample form Crume et al. (2025), which consisted of 30 college students enrolled in a ASL program at Georgia State University. All participants in that study were native users of English; However, two participants were raised by deaf Parents, but no information was available regarding their exposure to ASL. The mean age of that sample was 26.93 years. No other demographic information was reported for that sample.

The sample size for the present study was determined based on the practical constraints of collecting data from this population. It is a significant challenge to recruit adult Deaf participants who meet this criterion in a single metropolitan area, in part because historically Deaf individuals in the United States were not exposed to ASL in the educational system (Hall, 2017). We conducted a *post hoc* power analysis to provide the reader with an estimate of the power that the sample afforded to answer the research questions, given the observed segmentation judgment patterns, as a sensitivity analysis. Using data from Crume et al. (2025), a power analysis was conducted via simulation of a logistic mixed-effects model predicting event segmentation button press locations. Following Crume et al. (2025), we ran 1,000 simulations predicting segmentation judgments, binned via 1 s bins, from the situational change and the visual change variable scores, and their two population-by-predictor (ASL-learners vs. ASL-native users) interactions. We sampled predictor values from the real dataset so that their distributions are realistic. The model assumed 20 participants, each making 10 segmentation judgments. We set the interaction effects, *a priori*, to a small/moderate value of 0.20 on the log-odds scale. The analyses showed that there is 98% power to detect a population X situation change score interaction (98% of the simulations produced a significant interaction), and 97% power to detect a population X visual change score interaction (97% of the simulations produced a significant interaction).

### 2.2 Materials

The same materials used in Crume et al. (2025) were used in the present study, which consisted of six short folktales ranging from 22 to 42 sentences in length for the original English versions. Two video recordings were made for each story. The first version was an ASL adaptation of the original text versions of narratives created by a Deaf native user of ASL. The second consisted of the original text versions that were orally conveyed and subtitled,^2^ but as conveyed above, the data from these were not used in the present study. Table 3 shows the run times and descriptive statistics for the stories regarding the situation dimension change and visual change scores. The presentation of the modalities was blocked such that participants saw three stories in one modality, and then three stories in the other modality. We could not run enough participants to fully counterbalance assignment of stories to modalities and order with six stories. As such, we randomly group stories such that *The Farmer’s Bride*, *Hands*, and *The Woman Who Had No Shadow* were grouped in a block and *Jessie James has a Big Heart*, *White Cap*, and *How to Fool a Cat* were grouped in the other block. The assignment of story blocks to modality and the order of the blocked modalities were counterbalanced. Within the blocks, the order of the stories was random. Following Crume et al. (2025), the ASL movies were broken into 1 s time bins, which was the unit of analysis in the linear mixed effects models.

**Table 3:** The run time, mean situation changes scores, and mean visual change scores of each story in English and ASL.

Story title	Run time English	Run time ASL	Mean situation change score	Mean visual change score
The Farmer’s Bride	2:33	3:27	0.34 (0.90)	0.03 (0.01)
Hands	2:17	2:17	0.31 (0.91)	0.04 (0.01)
Jessie James Has a Big Heart	1:51	2:22	0.25 (0.77)	0.03 (0.01)
White Cap	2:27	2:31	0.36 (1.05)	0.04 (0.01)
The Woman Who Had No Shadow	2:30	3:12	0.31 (1.0)	0.04 (0.01)
How to Fool a Cat	2:40	2:51	0.34 (0.94)	0.03 (0.01)

Mean situation change and visual change scores are based on computing a mean change per time bin per story.

#### 2.2.1 Situational change scores

Crume et al. (2025) conducted a situational continuity analysis (see also Magliano et al., 2012; Kurby and Zacks, 2012) of the stories that considered eight dimensions of situational relatedness (see Table 1). Each sentence from the English version and corresponding content from the ASL version were coded to determine if there was a change in the dimensions in the story content (0 = continuous, 1 = changed). As noted above, the situational continuity analysis had to be conducted independently for the English and ASL stories because they did not correspond exactly in content or the time in which information was conveyed (see Table 2). The first dimension was characters, which involved the introduction of new and reintroduction of characters who were not part of the current event. The second dimension was similar but involved non-character animals and significant inanimate objects. The third dimension was space, which involved whether there were changes to a new story location. The fourth dimension was time, which involved whether there was a temporal gap between events. The fifth dimension was a change in the characters’ goals which involved the introduction of a new goal, its completion, or circumstances leading to its cessation. The sixth dimension was the beginning or ending of character-character interactions (physical or dialogic). The seventh dimension was the beginning or ending of character-object interactions. The final dimension involved changes in the internal states of characters, which involved perceptual (e.g., seeing, hearing), cognitive (e.g., belief, knowledge), and affective states. However, in these stories changes time and space tended to co-occur. As such, a spatial–temporal dimension was created that indicated if their was a change in either space or time. Crume et al. (2025) created a composite score for the exploratory analyses described above that involved adding the number of these dimensions that changed within the 1 s time bin, which consisted of the situational change scores. The score could range from 0 to 1. Mean situation change scores for time bings per story can be seen in Table 3.

#### 2.2.2 Visual change scores

We used the visual change analysis of the movies presented in Crume et al. (2025). Briefly, we first extracted each frame from each movie and stored the RGB values of each pixel. Second, we computed the average amount of visual change, pixel-by-pixel, between each frame and its immediately preceding frame, using 3-dimensional Euclidean distances, in RGB space. Mean visual change scores for time bins per story can be seen in Table 3.

### 2.3 Procedure

Participants were run individually in the study sessions. These sessions were run at a quiet location that was of convenience to the participants. The experiment was run on a laptop computer using Eprime 3.0. All instructions were presented in written English. A native ASL user (i.e., the fourth author, J. T. Malone) translated the instructions below into ASL and a video recording of him conveying instructions was shown to participants:

*As you know, stories contain lots of situations. If you think about it, the plot of a story is made of a series of situations that change as the story unfolds. As events happen, they can change the situations that make up the story.*

*As you experience the stories, think of the situations that make up the story. We want you to indicate when you think there is a change in the situation. It is up to you to decide what things change the situations in the stories. You will make your decisions by pressing the space bar on the keyboard.*

There were two practice stories: one was presented in ASL and the other in spoken English, with Subtitles. However, given that the participants were Deaf, there was no audio. After completing the practice stories, participants were allowed to ask the experimenter any remaining questions about the study. After each story, a prompt occurred that asked the participants to recall the stories in as much detail as they could remember, and they did so by using ASL and their responses were video recorded. The recall data will not be used in the present study because it is not relevant to the goal to explore the sensitivity to situational and visual change for the ASL versions.

### 2.4 Data analytic plan

As noted above, only the event segmentation data for the ASL versions of the stories were used in this study. For all analyses below, for each 1 s time bin of the movie, we recorded whether or not each participant pressed the button in that location and coded it as a binary variable (1 = yes, 0 = no). To address our research questions, we computed logistic mixed effect models predicting these segmentation judgments from fixed effects relevant for each research question. Particulars of the fixed effects are described below. For each model, participants and story were included as random effects. We used the lme4 library in R (version 4.5.0) (Bates et al., 2015) to run the models and estimate *p*-values, and we used the bobyqa optimizer.

## 3 Results

Participants produced a mean of 16.4 (*SD* = 11.9) event segmentation judgments per story. From these data, we computed average unit lengths by taking the length of the movie in seconds and dividing by the number of button presses plus one, which indicates the average length of segmented events. Mean unit length was 13.1 s(7.2 s) for the ASL story versions. As a preliminary analysis, we computed the level of agreement in the segmentation agreement using methods outlined by Kurby and Zacks (2012). The analysis assesses the degree of temporal association between each participant’s button presses and the group as a whole, scaled by the participant’s number of button presses. The mean level of segmentation agreement for the ASL story versions was 0.67 (*SD* = 0.08). This is comparable to the level of agreement for the ASL learners when segmenting ASL versions of the stories from Crume et al. (2025), which was a mean of 0.63 (*SD* = 0.02).

To address RQ1, consistent with Crume et al. (2025), we ran a logistic mixed effect model predicting whether there was a segmentation judgment in each time bin, from the situational change and the visual change scores. Participants and story topics served as random effects. As can be seen in Table 4, situational change scores were significantly, and positively, associated with event segmentation. Visual change was not significantly associated with event segmentation. These results show that segmentation judgments were associated with situational changes, but not with visual change scores for native signers.

**Table 4:** Logistic mixed effect results predicting segmentation location from total situational change and visual change scores for the ASL stories.

Fixed effects	*Estimate*	*SE*	*z*	*p*
(Intercept)	−2.48	0.16	−15.78	< 0.001***
Situational change	0.08	0.04	2.33	0.020*
Visual change	0.05	0.04	1.27	0.206

Random effects	Var	*SD*
Subject	0.44	0.67
Story	0.001	0.04

*R^2^_m_*	*R^2^_c_*
0.002	0.121

*p < 0.05; ***p < 0.001.

To address RQ2, the model from RQ1 was compared to a model that was identical to that for RQ1, but with the addition of an interaction between visual change and situational change scores. Results indicated that the addition of the interaction term did not improve fit over the RQ1 model, *Χ^2^(1)* = 0.05, *p* = 0.83. These results show that visual change did not moderate the relation between situational change and segmentation.

To address RQ3, we ran a new logistic mixed effects model that included the main effects of visual change, situational change, ASL language group (Native signers versus ASL leaners), the two-way interaction between visual and situational change, the two-way interactions between ASL language group and situational change, and the two-way interaction between ASL language group and visual change. We first asked whether the relation between visual change and segmentation was moderated by ASL language group. We initially tested a new main effects model that included ASL language group as a predictor of segmentation but did not include the two-way interactions with visual change and situational change. As can be seen in Table 5, for ASL language group, native ASL users were significantly more likely to perceive event boundaries in the stories than ASL learners. Also, as reported in Table 5, the interaction between ASL language group and visual change was significant. To investigate this interaction, slopes for the visual change predictor were extracted, separately for the native ASL users and ASL learners (ASL first language: *coef* = 0.05, *SE* = 0.04; ASL second language: *coef* = −0.08, *SE* = 0.04). Simple slope tests against zero indicated that the slope for ASL first language users was not significant, *z* = 1.41, *p* = 0.158, but it was significant for the ASL second language users, *z* = −2.16, *p* = 0.031. These results show that the effect of visual change on segmentation was moderated by ASL language group. For the situational change by ASL language group interaction, Table 5 shows that the interaction was not significant. Given that the effect of situational change was significant for ASL first language users, and Crume et al. (2025) reported that it was not significant for ASL second language users, this lack of interaction may be due to a lack of power. To investigate, similar to the analysis above, we estimated slopes of situational changes for each language group (ASL first language: *coef* = 0.08, *SE* = 0.04 ASL second language: *coef* = 0.03, *SE* = 0.04), and tested them against zero. The tests indicated that the slope for ASL first language users was significant (*z* = 2.34, *p* = 0.019) and the slope for ASL second language users was not (*z* = 0.79, *p* = 0.429). These results provide supportive evidence that the effect of situational changes on segmentation was moderated by ASL language group.

**Table 5:** Logistic mixed effect results predicting segmentation location from total situational changes, visual change, and ASL language group.

Fixed effects	*Estimate*	*SE*	*z*	*p*
(Intercept)	−2.89	0.12	−23.17	< 0.001***
Total changes	0.03	0.04	0.79	0.429
Visual change	−0.07	0.04	−1.75	0.080
ASL language group	0.42	0.18	2.28	0.022*
Total changes × visual change	−0.04	0.02	−1.77	0.077
Total changes × ASL group	0.05	0.05	1.01	0.313
Visual change × ASL group	0.13	0.05	2.53	0.012*

Random Effects	*Var*	*SD*
Subject	0.35	0.59
Story	0.01	0.10

*R^2^_m_*	*R^2^_c_*
0.015	0.111

*p < 0.05; ***p < 0.001. For ASL Language Group, ASL second-language served as the reference condition.

## 4 Discussion

Events are conveyed differently in spoken and signed languages, and these differences arguably create unique challenges for second language learners of signed languages. These differences create challenges in visual attention (Emmorey et al., 2009), and if learners do not attend to the articulators that convey important event information (Crume et al., 2025). The importance of research addressing these challenges is underscored by the fact that ASL is now the third most popular second language in postsecondary institutions in the United States (Robinson and Henner, 2018). A contingent of students taking these courses intend to enter professions in which translating ASL into spoken languages is required. As such, understanding the nature of these challenges is important to support second language learners of ASL. The present study should be construed as an important follow-up study to Crume et al. (2025) who presented data consistent with the hypothesis that learners of signed languages may overly attend to manual signs and miss other important articulators.

In the present study we explored three research questions involving data from native signers and data from ASL learners obtained from Crume et al. (2025). RQ1 addressed whether segmentation judgments for native signers were sensitive to situational and visual change scores. Consistent with our hypotheses, segmentation judgments predicted situational change, but not visual change. With respect to RQ2, we did not find that the relationship between situational change scores and segmentation judgments were moderated by visual change scores for natives signers. The analysis that addressed RQ3 confirmed that sensitivity to situational change and visual change was moderated by language status. The relationship between situational change and segmentation judgments was stronger for native signers than ASL learner, whereas the relationship between visual change and segmentation judgments was stronger for ASL learners than native signers.

The results of the present study extend those of Crume et al. (2025). Specifically, they suggest that native signers and learners of ASL may attend articulators used to convey events in ASL differently. While we argue the differences in the relationship between the visual change scores and segmentation judgments across native signers and ASL learners are likely due to the later attending to hand movement more than the former, it is important to remind the reader that Emmorey et al. (2009) did not find evidence for this difference using eye tracking data. They found differences in visual attention on the face, such that native signers attended to the eyes of a signer, whereas ASL learners attended to the movement of the mouth native users attended more to the eyes, whereas learners attended to the mouth. As such, both the present study and Emmorey et al. (2009) may suggest that learners attend to articulars that show relatively high amounts of motion during signing than more subtle articulators. Consistent with Emmorey et al. (2009), we assume that this is the case based on a misunderstanding of the importance of some of the articulators used in sign language, and the fact that spans of perception when processing ASL are likely larger for native signers than learners. Consequently, native signers can process some articulators accurately enough to support comprehension in peripheral vision. It is also important to note that we cannot compare the developmental status of acquiring ASL across the two studies, and it may be the case that Emmorey’s participants were at a higher state of proficiency in ASL than those from Crume et al. (2025).

It is important to acknowledge that event information is conveyed in the movement of signers’ hands for individual signs (and in particular verbs) (Krebs et al., 2021, 2026), and how those signs are sequenced (Krebs and Wilbur, 2024). For example, Krebs and Wilbur (2024) showed that there are differences in the word order of signs for intentional and unintentional events in Austrian Sign Language. As such, it is important to underscore that learners of signed languages do need to attend to the signs but may need additional support in learning to attend to other articulators as well.

To that end, the present study has implications for ASL instruction in postsecondary context. ASL language degree programs are typically taught as a sequence of courses (e.g., ASL 1, ASL 2, etc.). There are differences in the use of static materials (images, workbooks) versus dynamic materials (videos) and social/professional events (attendance at Deaf events, observation and practicum experiences with working interpreters) across the sequences. Specifically, early courses in a sequence tend to rely more on static materials with later courses relying on dynamic materials and experiences. This is typically due to the need for learner proficiencies to develop before dynamic experiences become more accessible to them. The demands on attentional selection different between static and dynamic materials (Loschky et al., 2026), which would have implications on how effective learners of ASL would be at attending to multiple articulators in a dynamic signing space. The results of the present study suggest that developing interventions that provide scaffolds to support attentional selection when courses transition to dynamic materials is warranted. More research is needed to determine how to develop such scaffolds.

This study illustrates the importance of interdisciplinary research on the study of the cognition of processing signed languages (e.g., Crume et al., 2025). The research team is made up of Deaf literacy researchers, ASL acquisition researchers and instructors, and cognitive scientists who study event cognition. Additionally, we hope that the present study motivates researchers in the psychological sciences that study event cognition to conduct research in this area. We believe that theories can help inform this area of research, but it also provides fertile ground for testing our theories. Moreover, event cognition researchers could contribute to understanding the challenges of learning ASL and the challenges for native signers in understanding events conveyed in second languages that are so different in how events are conveyed.

One such theory that could contribute to this area of research is the Event Cognition and Scene Perception Theory (SPECT) (Loschky et al., 2026). SPECT explains how perception, visual attention, and event comprehension are coordinated when people process visual events (e.g., movies). However, processing ASL similarly requires the coordination of these processes. SPECT specifically explains when visual attention is affected by stimulus factors, such as movement and one’s understanding of an event. The results of the present study suggest that the impact of stimulus features and comprehension on visual attention may change as one becomes proficient in signed languages. Specifically, when one is learning a signed language, visual attention is likely to be more heavily influenced by stimulus features, whereas as proficiency increases, comprehension will play a greater influence. More research is needed to explore this possibility and SPECT could provide a theoretical framework to guide such research.

It is also important to acknowledge that the fourth (a CODA) and fifth (a Deaf individual) authors are native signers. Collins et al. (2018) has argued that research involving specific populations (autistic individuals, Deaf individuals) should include members of those populations as part of the research team when possible. Some scholars who study Deaf populations have argued that doing so is an essential aspect of ethical research (Krawczyk et al., 2024; Singleton et al., 2014). Inclusion of such Deaf individuals ensures accurate understanding of Deaf participants’ responses from a native language user and a culturally appropriate and aware approach to data collection, analyses, and the dissemination of the findings. While we recognize that it is not always possible to include deaf individuals on a research team, we can attest to the positive impact that it had on this collaboration.

There are some limitations of the present study that should be acknowledged. First, the sample of natives signers is relatively small for a study of this nature. This is often typical of studies involving natives signers because it is typically very challenging to recruit participants without resources to recruit regionally or nationally. Additionally, our sample consisted of Deaf and Hearing individuals. The small sample size did not afford exploring if there are differences between Deaf and hearing native signers. Nonetheless, our results clearly confirmed our hypotheses associated with our research questions. Second, we did not have measures of ASL proficiency. While all our participants met our requirements for native signers, there are individual differences in ASL proficiency as is the case with native users of spoken languages (Lillo-Martin et al., 2000). Third, we used videos of one individual conveying the stories. Other researchers have the stories conveyed to participants in real time (Emmorey et al., 2009). It may be the case that learners of ASL are more sensitive to multiple articulators when viewing signers in real time. Finally, ASL is not a universal language, and across the globe there are scores of other signed languages used in Deaf communities. Moreover, there are different dialects of signed languages, and there are differences across dialects in the signing space ASL [e.g., Black ASL (BASL) generally uses a larger signing space than standard ASL; Hill et al., 2015; McCaskill et al., 2011]. It is important to verify if findings in one signing system are replicated in other signing systems (Krebs and Wilbur, 2024). Differences in signing space across sign languages may have implications on the visual attentional demands. Future research on the attentional demands of learning signed languages should focus on other signed languages and dialects within those languages.

It is also important to acknowledge that the event segmentation task could have differentially affected how participants attended to articulators. The event segmentation task is relatively naturalistic and easy to perform, and there is evidence that the transient changes in networks of the brain that support event comprehension and segmentation are similar when segmenting events and watching them passively (Zacks et al., 2001). However, most of these studies use videos of people performing everyday actions, and as such participants can draw upon extensive background knowledge to support processing the events. As such, the event segmentation task does not affect event comprehension in these contexts. Native signers are likely less affected by the task than ASL learners. Future research should compare event comprehension in the context of a segmentation task and outside of that context for native users and learners of ASL when processing events in ASL and English.

Additionally, event segmentation studies often require participants to segment and fine and coarse grain sizes (e.g., Zacks et al., 2009). When segmenting at a fine grain size, participants consider the smallest meaningful events when making judgments and when segmenting on a coarse grain size, they consider the largest meaningful events. Having participants segment on fine and coarse grain sizes enables assessing how smaller events are nested within larger events, which affords studying the extent that people are sensitive to the hierarchical structure of events (Kurby and Zacks, 2012). However, the short length of the stories did not afford having participants segment at the two grain sizes. Future research should use longer narratives that afford segmenting at multiple grain sizes; doing so would afford assessing how multiple articulators convey the hierarchical structure of events in signed languages.

Studies that have assessed the relationship between situational change and event segmentation have typically explored the relationship between individual situational dimensions (e.g., time, space, characters, causality, etc) segmentation judgments (e.g., Kurby and Zacks, 2012; Magliano et al., 2012; Zacks et al., 2009). Unfortunately, the sample size of the current did not afford exploring those relationships because there was insufficient statistical power to do so. While it would be challenging to conduct a study with deaf participants that would have a sample size that would afford those analyses, it would be worthwhile to do so in future studies.

The visual change scores reflect all the movement occurring in the videos, and therefore reflects movement associated with manual and non-manual articulators. Future research could combine eye tracking and event segmentation judgments. This would allow a finer grain assessment of how visual attention to manual and non-manual articulators is related to event comprehension in learners and native users of signed languages.

Finally, as noted the present study made use of only part of the data that was collected. The data collection replicated that of Crume et al. (2025), except that the English stories were presented with subtitles rather than orally conveyed. A future study will make use of all the data, which will allow an assessment of the sensitivity to situational change changes as a function of whether the stories were conveyed in the participants native language or in their second language. Crume et al. (2025) showed that learners were more sensitive to situational changes when the stories were presented in English than ASL. It remains to be seen as to whether the opposite will be the case for adult, Deaf native signers.

## 5 Conclusion

Given the increase in the popularity of ASL as a second language and the fact that many students seek professions that require ASL, research that helps us understand ASL learners is of paramount importance. In order to become effective teachers of Deaf students or interpreters for Deaf clients, hearing learners of ASL need to become highly fluent in the language. Without high degrees of fluency, we risk employing hearing individuals in Deaf community-facing professions who may unintentionally cause harm to their clients because their ASL communication may be insufficient (Scott et al., 2023). It is essential that this line of research continues to grow so that instructors who are providing instruction to ASL learners can use this information to create the most effective learning environment possible. We believe that scholars who study event cognition can contribute to this effort because they are interested in the coordination of visual attention and comprehension, a significant challenge in learning signed languages. Our hope is that the present study motivates such research. However, we underscore the importance of ensuring that such research should involve interdisciplinary collaboration with Deaf literacy researchers and signed language instructors.

## Data Availability

The datasets presented in this study can be found in online repositories. The names of the repository/repositories and accession number(s) can be found below: https://zenodo.org/records/23103944.

## Footnotes

1 While the stories had a high amount of overlap in narrative events, there were differences between the versions as reflected in Table 1 and as such, separate situational dimension continuity analyses were performed on the ASL and English versions of the stories.

2 The videos were subtitled because the participants were Deaf. The subtitles reflect closed and open captioning that Deaf and hard of hearing individuals rely upon to process orally conveyed spoken languages.
